# Biodegradation of Crude Oil and Corexit 9500 in Arctic Seawater

**DOI:** 10.3389/fmicb.2018.01788

**Published:** 2018-08-06

**Authors:** Kelly M. McFarlin, Matt J. Perkins, Jennifer A. Field, Mary B. Leigh

**Affiliations:** ^1^Institute of Arctic Biology, University of Alaska Fairbanks, Fairbanks, AK, United States; ^2^Department of Environmental and Molecular Toxicology, Oregon State University, Corvallis, OR, United States

**Keywords:** Arctic seawater, biodegradation, crude oil pollution, Corexit, GeoChip, 16S rRNA gene sequencing

## Abstract

The need to understand the biodegradation of oil and chemical dispersants in Arctic marine environments is increasing alongside growth in oil exploration and transport in the region. We chemically quantified biodegradation and abiotic losses of crude oil and Corexit 9500, when present separately, in incubations of Arctic seawater and identified microorganisms potentially involved in biodegradation of these substrates based on shifts in bacterial community structure (16S rRNA genes) and abundance of biodegradation genes (GeoChip 5.0 microarray). Incubations were performed over 28-day time courses using surface seawater collected from near-shore and offshore locations in the Chukchi Sea. Within 28 days, the indigenous microbial community biodegraded 36% (*k* = 0.010 day^-1^) and 41% (*k* = 0.014 day^-1^) of oil and biodegraded 77% and 33% (*k* = 0.015 day^-1^) of the Corexit 9500 component dioctyl sodium sulfosuccinate (DOSS) in respective near-shore and offshore incubations. Non-ionic surfactants (Span 80, Tween 80, and Tween 85) present in Corexit 9500 were non-detectable by 28 days due to a combination of abiotic losses and biodegradation. Microorganisms utilized oil and Corexit 9500 as growth substrates during the incubation, with the Corexit 9500 stimulating more extensive growth than oil within 28 days. Taxa known to include oil-degrading bacteria (e.g., *Oleispira*, *Polaribacter*, and *Colwellia*) and some oil biodegradation genes (e.g., *alkB*, *nagG*, and *pchCF*) increased in relative abundance in response to both oil and Corexit 9500. These results increase our understanding of oil and dispersant biodegradation in the Arctic and suggest that some bacteria may be capable of biodegrading both oil and Corexit 9500.

## Introduction

As Arctic sea ice cover retreats due to climate change (Comiso et al., 2008), there has been an increase in oil exploration and shipping traffic (National Research Council [NRC], 2014), both of which increase the likelihood of oil spills (Bureau of Ocean Energy Management [BOEM], 2015). This increases the need to improve our understanding of the biodegradation of crude oil and oil spill response chemicals, such as chemical dispersants, in the Arctic marine environment.

A variety of dispersant formulations have been applied to oil spills worldwide (National Research Council [NRC], 2005; Chapman et al., 2007), with the most substantial application being that of Corexit 9500 in the Gulf of Mexico (Deepwater Horizon oil spill, >43,000 barrels; National Commission, 2010). When applied to a surface oil slick, surfactant compounds within dispersants reduce the surface tension between water and oil (Rouse et al., 1994), allowing the oil to become mixed into the water column as tiny droplets (Camilli et al., 2010; Brakstad et al., 2014). The creation of these droplets has been shown to significantly increase oil biodegradation (Brakstad et al., 2014, 2015; Prince and Butler, 2014). Corexit 9500 has been reported to be less toxic to several key Arctic marine species than oil (Gardiner et al., 2013); however, negative impacts of prior Corexit formulations (i.e., 9527) have been reported (Bruheim et al., 1999), and dispersant use in general adds more chemicals to the environment which may have unintended consequences. Thus, Corexit 9500’s biodegradation and impacts on microorganisms in Arctic seawater are of interest in order to better understand its potential fate and effects in the environment.

If an oil spill occurs in the Arctic, Corexit 9500 will likely be the dispersant of choice due to its efficacy (SL Ross Environmental Research Ltd and MAR Inc et al., 2007; Belore et al., 2009), relatively low toxicity (US EPA, 2010), reported effectiveness during the Deepwater Horizon (DWH) oil spill (Bejarano et al., 2013), and prior approval for use in subarctic Alaskan waters (AlaskaRegional Response Team [ARRT], 2004). The primary components of Corexit 9500 are the ionic surfactant DOSS (dioctyl sodium sulfosuccinate; 18% w/w; Place et al., 2016), the non-ionic surfactants Span 80, Tween 80, and Tween 85 (27% w/w; Place et al., 2016), and the carrier solvents dipropylene glycol butyl ether and petroleum distillates (∼55% w/w; National Research Council [NRC], 2005; Ramirez et al., 2013; Parker et al., 2014).

The mineralization of Corexit 9500 (based on respirometry) and the extent of crude oil biodegradation in Arctic seawater have been reported (McFarlin et al., 2014), although the rates at which Corexit 9500 and oil degrade in Arctic environments are still unknown (National Research Council [NRC], 2014). Corexit biodegradation studies have been conducted with Gulf of Mexico seawater (Campo et al., 2013; Kleindienst et al., 2015; Seidel et al., 2016; Techtmann et al., 2017) at low temperatures (5 and 8°C), but the metabolic functions of these temperate deep-sea microbial communities may not be representative of Arctic communities.

Rates of oil biodegradation are generally thought to be slower in Arctic than temperate regions (Margesin et al., 2003; Michaud et al., 2004) due to the influence of temperature on metabolic processes (Mihelcic, 1999); however, some microorganisms are adapted to low temperatures (Feller, 2003), which may explain why some have reported similar oil biodegradation rates in cold and temperate environments (Braddock and McCarthy, 1996; Margesin and Schinner, 1997; Gibb et al., 2001). Previous studies have demonstrated the ability of indigenous Arctic microorganisms to biodegrade oil (Atlas et al., 1978; McFarlin et al., 2014; Garneau et al., 2016), although data gaps remain regarding the taxonomic identity of Arctic oil-degraders in seawater. Experiments with indigenous microbial communities have been conducted with Arctic sea ice (Gerdes et al., 2005; Brakstad et al., 2008; Garneau et al., 2016), but what has been reported for seawater relies primarily on culture-based methods that may be taxonomically and functionally unrepresentative of the sampled environment. Such studies have, however, indicated that a variety of bacterial taxa (e.g., *Marinobacter*, *Pseudoalteromonas*, *Colwellia*, *Oleispira*, and *Psychrobacter*) are associated with oil biodegradation in Arctic seawater and sea ice (Yakimov et al., 2003, 2007; Deppe et al., 2005). Some of these taxa, including *Colwellia*, were also enriched by chemically dispersed oil in a cold deep-water plume during the DWH oil spill (Redmond and Valentine, 2012; Dubinsky et al., 2013). The increased abundance of *Colwellia*, a known oil-degrading taxon (Dubinsky et al., 2013), in experimental incubations containing Corexit 9500-only and chemically dispersed oil suggests that members within this genus may be able to degrade Corexit in addition to oil (Brakstad et al., 2015; Kleindienst et al., 2015).

It is likely that microorganisms capable of degrading oil are also capable of degrading some components of dispersants due to similarities in their chemical composition. Crude oil generally consists of hydrocarbons classified as alkanes (linear and branched; 33% by wt.), cycloalkanes (32%), and aromatics (35%) (Tissot and Welte, 1984). The petroleum distillate (CAS# 64742-47-8) fraction of Corexit 9500 also consists of hydrocarbons (C_9_ – C_16_), including C_10_ cycloalkanes, and *iso*- and *n*-paraffins (Petroleum Distillates SDS, 2012). In addition to the hydrocarbons present in the carrier solvent fraction of Corexit, hydrocarbon side chains are also present in DOSS (Seidel et al., 2016), and the non-ionic surfactants (NALCO, 2008; Word et al., 2015). Since hydrocarbons are found in both oil and Corexit 9500, similar taxa and similar functional genes may be involved in their biodegradation.

Here, we combine chemical analyses with microbial analyses to characterize the biodegradation of oil and Corexit, separately, in Arctic seawater mesocosms. We report biodegradation extents and/or rates of crude oil and selected Corexit components in Arctic seawater and make comparisons between near-shore and offshore seawater. We also identify microbial taxa and biodegradation genes that increased in response to oil or Corexit. We found that some of the same taxa and oil biodegradation genes (e.g., *alkB*) increased in relative abundance in response to both oil and Corexit, suggesting that some organisms may be capable of biodegrading components of both.

## Materials and Methods

### Seawater Collection and Nutrient Addition

Three separate batches of surface seawater were collected. Two offshore samples were collected separately from the Burger oil lease area (∼90 km from Wainwright, AK, United States) in September and October 2013 (71.1050° N, -162.2668° W; hereafter referred to as offshore), and one near-shore sample from ∼1 km from Utqiagvik (formerly named Barrow), Alaska (71.3647° N, -156.5241° W) in August 2014. Surface seawater temperature was 2°C upon collection. Samples were held at 2°C onboard the vessel and transported in coolers with icepacks to the University of Alaska Fairbanks within 24 h. Upon receipt, seawater was placed in a cold room (2°C), aerated, and amended with a small amount of nutrients (0.01635 g/L of Bushnell Haas broth; Bushnell and Haas, 1941). Bushnell Haas (BH) broth is a common nutrient addition to oil biodegradation experiments conducted in closed systems. In powdered form, the BH was added to the initial seawater prior to distribution into individual mesocosms. While other incubation experiments have added the full recommended volume of BH (Lindstrom and Braddock, 2002) or similar nutrient concentrations and formulations (GP2; Venosa and Holder, 2007), we added 0.5% of the recommended volume of BH (equal to 16 mg/L) to mimic natural conditions as much as possible. These nutrients added 1 mg/L magnesium sulfate (MgSO_4_), 5 mg/L monopotassium phosphate (KH_2_PO_4_), 5 mg/L dipotassium phosphate (K_2_HPO_4_), 5 mg/L ammonium nitrate (NH_4_NO_3_), and 0.25 mg/L ferric chloride (FeCl_3_). These nutrient amendments are within one order of magnitude (or less) of background nutrient concentrations reported for Arctic seawater (Codispoti et al., 2005; McFarlin et al., 2017). All incubations contained this nutrient-amended seawater, including the abiotic and biotic controls for chemical and microbial analyses. Our prior studies found similar oil loss in 60 days with this nutrient amendment compared to unamended incubations, with the latter being larger in volume and lower in oil concentration with the intent of mitigating nutrient limitations (McFarlin et al., 2014). Within 48 h of seawater collection, including 24 h of aeration, biodegradation incubation experiments were initiated.

### Oil and Corexit 9500

Alaska North Slope (ANS) crude oil was obtained from the Alyeska pipeline terminal in Valdez, AK, United States (Summer, 2009). The oil was not deliberately weathered prior to addition, but prior to the start of this experiment in 2013 the oil may have undergone some natural weathering. Corexit 9500 was obtained from Nalco Inc. (Naperville Township, IL, United States), during the summer of 2009. Positive displacement pipettes were used to accurately add defined volumes of oil or Corexit.

### Incubation Studies

Incubations were conducted in 1-L small-mouth glass bottles, with caps tilted to allow air exchange. Each mesocosm contained 800 mL of Arctic surface seawater, and either ANS crude oil (15 mg/L) or Corexit 9500 (15 mg/L) directly added to the surface of the seawater. As a control, we also incubated seawater in the absence of oil or Corexit to reveal bottle-associated microbial community shifts. Incubations were constantly stirred using magnetic stir bars (Teflon coated; no vortex) at 2°C in a cold room with an 8-h/day light cycle (8.88 μmol s^-1^ m^-2^; LI-COR light meter, LI-250, Quantum Sensor, Lincoln, NE, United States). Replicate mesocosms were destructively harvested over a time course (0, 5, 10, and 28 days) for chemical analyses (*n* = 3) and microbial analyses (*n* = 3), although in some cases (e.g., a subset of October offshore incubations), mesocosms were only harvested in duplicates or fewer time points were used due to the limited quantity of collected seawater (see **Supplementary Table S1** for a summary of the experimental design). Upon harvesting, mesocosms designated for microbial analyses were filtered (0.2 μm) and the filters were frozen (-80°C) until DNA extraction, while the mesocosms destined for chemical analyses were frozen at -20°C until extraction.

### Chemical Analysis of Crude Oil

The chemical loss of total extractable ANS crude oil was determined in both biotic and abiotic control incubations. The entire contents of each flask were extracted and analyzed for total petroleum hydrocarbons (TPHs) by gas chromatography-flame ionization detector (GC-FID), and individual alkanes and aromatics by GC/MS-selected ion monitoring (SIM) (B&B Laboratories, College Station, TX, United States). We normalized concentrations to 17α(H), 21β(H)-hopane as a conserved internal marker (Prince et al., 1994) and determined total oil biodegradation as percent loss relative to time zero. Abiotic losses were measured in killed (autoclaved seawater) controls and were subtracted from all time points to calculate biodegradation. Using first-order kinetics (Mihelcic, 1999), we used the rate law for a first order reaction ([C] = [C_0_]e^-^*^k^*^t^) to calculate the biodegradation rate constant (*k*) (Stewart et al., 1993; Venosa and Holder, 2007; Brakstad et al., 2008).

### Chemical Analysis of Corexit 9500

At each time point, incubation bottles were immediately diluted to 75% with isopropanol (seawater:isopropanol, 75:25), and stored at -20°C. Aliquots (50 mL) were shipped overnight on dry ice to Oregon State University and stored at -20°C until analysis. Aliquots were diluted (0- to 100-fold) with a combination of instant ocean and isopropanol (75:25), and quantitative analysis of surfactant components was performed as described by Place et al. (2016), with minor modifications. Liquid chromatography mass spectrometric detection was performed with a Waters Micromass Quattro Mass Spectrometer as described previously (Place et al., 2016). We used an Agilent Poroshell 120 EC-C18 guard column (4.6 mm ID × 5 mm length, with 2.7-μm particles) to accommodate high backpressure. A 50 mm Targa C18 analytical column (2.1 mm ID × 50 mm, with 5-mm particles; Higgins Analytical, Inc., Mountain View, CA, United States) was used for chromatographic separations. The 50-mm column allowed for the flow rate to be increased to 1 mL/min during sample loading and washing non-volatile salts from the column (first 5.6 min) without degrading peak shape or percent recovery of analytes. The gradient was further modified such that the 97.5% acetonitrile was held for 3 min before returning to 5% acetonitrile for 6 min. The flow rate was 1 mL/min for the first 6 min, 0.5 mL/min for the next 5 min, and 1.0 mL/min for the last 6 min. The timing of the main-pass by-pass valve switching and divert valve switching, as described by Place et al. (2016), was adjusted to reflect changes in the flow rate and gradient.

Calibration curves consisted of at least five calibration standards and required a correlation coefficient of 0.99 or greater for use in analysis. All calibration curves were weighted (factor of 1/x), and standards with calculated concentrations above 20% of intended concentrations were removed from the calibration curve calculation. Calibration curves spanned from the lower limit of quantification (LLOQ) to the upper limit of quantification (ULOQ): for DOSS (0.2–25 μg/L), Span 80 (60–300 μg/L), Tween 80 (60–300 μg/L), and Tween 85 (60–300 μg/L), and samples were diluted to concentrations within this range. Each calibration standard was spiked to give a final concentration of 500 ng/L ^13^C4-DOSS. Blank and check standards, as described by Place et al. (2016), were used for quality control purposes and consisted of at least 20% of the total samples run in any given sequence. Check standards for DOSS fell within 20% of the spiked concentration and the non-ionic Corexit surfactants fell within 35% of the spiked concentration. All blanks fell below the limit of detection. The limits of detection (LOD) for all analytes were determined as described in Place et al. (2016), with DOSS having a LOD of 0.08 μg/L, Span 80 having a LOD of 5.5 μg/L, Tween 80 having a LOD of 15 μg/L, and Tween 85 having a LOD of 6.5 μg/L. The LOD for DOSS was similar to that reported by Place et al. (2016), while the LOD for the non-ionic surfactants was greater by a factor of 5–15. Different instruments and chromatographic conditions, as described above, were used in our analysis and could explain these differences.

### Quantitative Real-Time PCR

Quantitative real-time polymerase chain reaction (q-PCR) targeting 16S rRNA genes was used to quantify prokaryotic populations in all treatments (i.e., biotic and abiotic, *n* = 3). Results are reported as copies of 16S rRNA gene sequences per 800 mL of seawater (i.e., per incubation). Briefly, a double-stranded DNA molecule of 482 bp spanning the V4-V5 region was synthesized (IDT, Coralville, IA, United States) and re-suspended to known molarity for use as a 16S rRNA qPCR standard. PCR oligonucleotide primers were also synthesized complementary to the 5′ and 3′ region of the synthesized gBlock fragment, and designed based on known prokaryotic 16S rRNA gene sequences. The primers were GTGCCAGCMGCCGCGGTAA (“515F Original,” Caporaso et al., 2010; Walters et al., 2016) and GGACTACNVGGGTWTCTAAT (“806R Modified,” Apprill et al., 2015; Walters et al., 2016). All q-PCR reactions contained 7.5 μl of Power SYBR^®^ Green PCR Master Mix (Applied Biosystems, Grand Island, NY, United States), 0.4 μl (10 μM) of primer, 3.7 μl of PCR water, 3 μl of template DNA. A non-template control was also run. All samples, standards, and controls were run in triplicate on an ABI 7900HT Sequence Detection System (Life Technologies, Grand Island, NY, United States) using the parameters outlined in Martinez-Cruz et al. (2017). A regression line was created using the standard dilution series and accepted if *R*^2^ > 0.99. SDS (version 2.2.2; Life Technologies, Grand Island, NY, United States) was used to analyze the results.

### Microbial Community Analysis

We sequenced 16S rRNA genes to determine the taxonomic identity and relative abundance of bacteria in the October offshore and August near-shore incubations. We extracted total DNA from filters containing microorganisms from each treatment flask (Miller et al., 1999). The DNA extract was sequenced on Illumina’s MiSeq platform using indexed primers (F515/R806) that targeted the V4 region (Caporaso et al., 2012). PCR products were normalized and pooled using an Invitrogen SequalPrep DNA Normalization plate (Thermo Fisher Scientific, Waltham, MA, United States). The pooled libraries were quality controlled and quantified prior to loading on an Illumina MiSeq v2 flow cell and sequenced in a 2 × 250 bp format with a standard v2 500 cycle reagent cartridge. Base calling was done by Illumina Real Time Analysis (RTA) v1.18.54 and output of RTA was demultiplexed and converted to FastQ format with Illumina Bcl2fastq v1.8.4. We then analyzed DNA sequences with mothur open source software (Schloss et al., 2009) following the online standard operating procedure (Schloss et al., 2011) and determined the taxonomic identity of bacteria using the Ribosomal Database Project (Wang et al., 2007). Operational taxonomic units (OTUs) were defined at 97% similarity. After the removal of singletons, relative abundances were normalized to total abundance per sample. All stated increases in relative abundance were compared to the biotic controls at the same time point.

### Functional Gene Analysis

The GeoChip 5.0_108K (Glomics Inc., Norman, OK, United States), a functional gene microarray, was used to determine the presence and relative abundance of petroleum degradation genes in the October offshore incubation. Using an aliquot of the same original DNA extract used for 16S rRNA amplicon-based microbial community analysis (described above), Glomics, Inc. conducted GeoChip analysis, which included amplification, labeling, hybridization, and data preprocessing (Van Nostrand et al., 2016). GeoChips were imaged (NimbleGen MS 200 microarray scanner; Roche NimbleGen Inc., Madison, WI, United States) and the data were extracted using the Agilent Feature Extraction program. Extracted data were then loaded onto the GeoChip data analysis pipeline^1^ where singletons were removed. Prior to statistical analyses, all signals were converted into relative abundances.

### Statistical Analyses

Non-metric multidimensional scaling (NMS), clustering analysis, indicator species analysis (ISA), and multi-response permutation procedures (MRPPs) were all conducted with PC-ORD V6 (McCune and Mefford, 2011).

We used NMS ordination plots to illustrate differences in bacterial community structure and the abundance of petroleum biodegradation genes in treatments containing ANS crude oil, Corexit 9500A, and biotic (no oil or Corexit added) controls. The dimensionality of the data within each NMS was determined with a Bray–Curtis distance measure in autopilot mode using 100 runs with real data and random starting configurations (Kruskal, 1964; Mather, 1976). After the NMS was created, a Monte Carlo test with 249 randomized runs was conducted to evaluate whether the NMS was extracting stronger axes than expected by chance. The stability of each solution was determined by plotting stress versus iteration (McCune and Mefford, 2011).

Cluster analysis objectively identifies groups that are most similar and builds groups within groups to show differences. We used hierarchical clustering to determine similarities among treatments containing ANS crude oil, Corexit, and neither amendment. Hierarchical clustering was performed with a Bray–Curtis distance measure and similarities were illustrated in a dendrogram. The dendrogram was created with a group average linkage method and was not scaled or pruned.

Indicator species analysis is a statistical calculation that indicates which species are associated with particular treatments or other groups of samples. ISA was used to identify taxa that were associated with either ANS crude oil or Corexit in seawater incubations. This statistical analysis revealed organisms that responded to oil or dispersant by calculating the proportional abundance and consistency of a particular species in a treatment relative to that species in all other treatments (McCune and Grace, 2002). We conducted an ISA using the default Dufrêne and Legendre (1997) analysis. Results are reported as an indicator value (IV) for each species and the statistical significance of each IV was evaluated by a Monte Carlo method. Indicator values range from zero (no indication) to 100 (perfect indication). Species that had statistically significant *p*-values (*p* < 0.05) are reported with their IVs for oil and dispersant treatments at day 28.

Statistical differences were calculated using MRPP, a non-parametric procedure for testing differences among groups (Mielke, 1984; Mielke and Berry, 2001). We found the data to be non-normal using the Shapiro–Wilk test (*W* = 0.837, *p* = 0.053). Each MRPP was conducted using a Bray–Curtis distance measure. We compared the % loss of oil and Corexit among their respective time points and reported the *p*-value.

## Results

### Biodegradation of Crude Oil

The indigenous microorganisms biodegraded 16 ± 4% (mean ± SD) of ANS crude oil within the first 5 days in both offshore incubations (**Table 1**). The extent of oil biodegradation at 28 days was similar between the two offshore samples (collected in September and October), with extents ranging from 36 ± 6% to 41 ± 0.0% (**Table 1**; MRPP, *p* > 0.05). The rate constants (*k*) for the September and October offshore oil biodegradation experiments were 0.010 day^-1^ and 0.014 day^-1^, respectively.

**Table 1:** Mean percent loss of total measurable hydrocarbons in Arctic surface seawater.

Location	Reference	Oil (mg/L)	Temp (°C)	Nutrients (mg/L)	Percent loss			
					Day 5	Day 10	Day 28	Day 63
Offshore (September)	This study	15	2	16	16 ± 4.2^a^	29 ± 5.5^b^	36 ± 6.2^bc^	nm
Offshore (October)	This study	15	2	16	16 ± 4.6^a^	28 ± 3.1^b^	41 ± 0.02^c^	nm
Near-shore (February)	McFarlin et al., 2014	2.5	-1	0	nm	36 ± 3^∗^	45 ± 3.6^∗^	58 ± 10^∗^


*All incubations contained whole ANS crude oil (*n* = 3). Letters correspond to significant differences among time points (MRPP, *p* < 0.05). Nutrient medium consisted of Bushnell Haas broth (0.5% of recommended volume). Error bars are standard deviation. nm, not measured. ^∗^Percent losses includes abiotic losses.*

### Biodegradation of Corexit 9500 Components

The concentration of DOSS decreased over the course of the offshore and near-shore incubations to varying extents. DOSS concentrations significantly decreased between day 0 and 28 in the offshore (by 98%) and near-shore (by 35%) incubations. The abiotic controls showed losses ranging from 2 to 21%, which were not significantly different from initial DOSS concentrations (*p* > 0.05; **Table 2**). In order to provide a conservative measurement of DOSS biodegradation, we subtracted the abiotic loss from the mean at day 0 and report that DOSS biodegraded by 77% in offshore seawater and 33% in near-shore seawater. The near-shore incubation included both 10- and 28-day time points and indicated that substantial DOSS biodegradation (biotic minus abiotic) occurred within the first 10 days (24%; *p* > 0.05; **Table 2**) and continued through the final 28-day time point. Since the offshore incubation included two time points (day 0 and 28) and the near-shore incubation included three time points (day 0, 10, and 28), we used the near-shore incubation to calculate the rate constant (*k*) for DOSS, which was 0.015 day^-1^.

**Table 2:** Mean concentration, % loss, and % biodegraded (biodeg.) of the surfactant components of Corexit 9500 in offshore and near-shore seawater at 2°C (n = 3).

	DOSS			SPAN			Tweens		
Offshore	μg/L	% Loss	% Biodeg.	μg/L	% Loss	% Biodeg.	μg/L	% Loss	% Biodeg.
Day 0	2880 ± 697^a^			244 ± 30^e^			2010 ± 20^q^		
Day 28A	2270 ± 400^a^	21		132 ± 91^efg^	46		1230 ± 570^q^	39	
Day 28	71 ± 15^d^	98	77	<LOD	>98	52–54	<LOD	>99	60–61
Near-shore									
Day 0	2260 ± 210^a^			179 ± 23^efg^			2240 ± 110^r^		
Day 10A	2190 ± 120^a^	3		150 ± 10^f^	16		409 ± 33^s^	82	
Day 10	1660 ± 200^b^	27	24	44 ± 2^h^	75	59	<LOD	>99	17–18
Day 28A	2210 ± 220^a^	2		177 ± 8^g^	1		298 ± 41^t^	87	
Day 28	1460 ± 170^c^	35	33	<LOD	>97	96–99	<LOD	>99	12–13


*Percent loss is relative to time zero and based upon mean values. Bottles contained seawater (800 mL), nutrients (16 mg/L Bushnell Haas Broth), and Corexit (15 mg/L). Abiotic controls are designated with an ‘A’ after the time point. Different letters correspond to significant differences within each surfactant (MRPP, p < 0.05). Errors represent standard deviations. The limit of detection (LOD) for Span 80, Tween 80, and Tween 85 was 5.5 μg/L, 15 μg/L, and 6.5 μg/L, respectively.*

Within 28 days, concentrations of the non-ionic surfactant Span 80 and the Tweens (Tween 80 and 85) dropped to below the LOD of 5 μg/L, 15 μg/L, and 6.5 μg/L, respectively (**Table 2**). Abiotic losses at day 10 and 28 were statistically significant for Tweens, yet greater losses were observed in biotic treatments (which dropped to below LOD; **Table 2**). For Span 80, abiotic losses were highly variable and not statistically significant (ranging from 1 to 46%) in 28 days, while losses in biotic treatments were significant, as evidenced by significantly lower concentrations at day 10 and 28 in biotic treatments compared to day 0 and abiotic controls. It should be noted that these % loss calculations (for oil and Corexit) are based upon mean values. Higher variability was observed for DOSS in the Corexit offshore experiment at day 0 than is observed for other time points or surfactants. This variability could be explained by errors in subsampling, extraction efficiency, or dilutions. When calculating biodegradation, we subtracted mean abiotic loss at each time point from mean values at day 0 and therefore assumed that compounds lost through abiotic means were never available for biodegradation. Therefore, the rates and extents reported here are likely conservative.

### Prokaryotic Population Size

**FIGURE 1:**
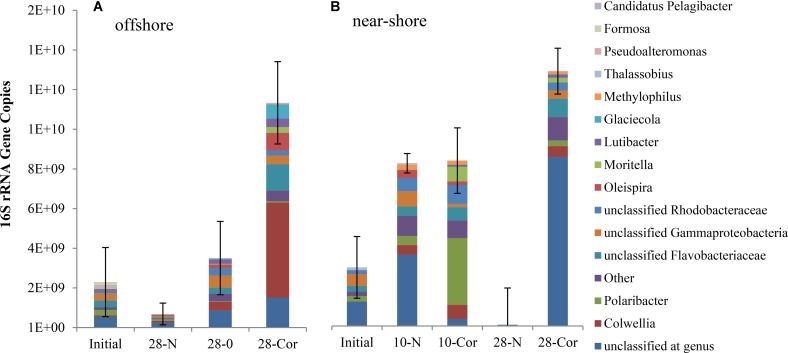
Mean relative abundance of bacterial taxa as a portion of mean prokaryotic abundance in experiments with **(A)** offshore and **(B)** near-shore seawater at 2°C (*n* = 3). Total 16S rRNA copies were determined by qPCR (per 800 mL incubation) and community structure was identified using 16S rRNA amplicon sequencing. Bottles contained surface seawater (800 mL), and either no amendment (N, biotic control), ANS crude oil (O; 15 mg/L), or Corexit (Cor; 15 mg/L).

Over the course of the offshore and near-shore incubations, we quantified prokaryotic (bacterial and archaeal) 16S rRNA gene copies using qPCR to determine if population growth occurred concurrently with the biodegradation of oil or Corexit. Total 16S rRNA gene copies increased in abundance in response to the presence of oil or Corexit within 28 days (**Figure 1** and **Supplementary Figure S1**). At 28 days, three-fold more 16S rRNA genes were present in offshore seawater with Corexit than with oil (1.1 × 10^10^ vs. 3.5 × 10^9^, **Figure 1** and **Supplementary Figure S1**); however, the Corexit treatment was more variable than the oil treatment. In near-shore seawater incubations containing no oil or Corexit addition, prokaryotes significantly (*p* > 0.05) increased in abundance between day 0 (3.3 × 10^9^ gene copies per 800 mL incubation = 4.1 × 10^9^ copies per L) and day 10 (9.2 × 10^9^ gene copies per 800 mL incubation = 1.2 × 10^10^ copies per L), but between day 10 and day 28 prokaryotes significantly decreased with only 2% of the initial abundance remaining at day 28 (7.0 × 10^7^ gene copies = 8.7 × 10^7^ copies per L) (**Figure 1** and **Supplementary Figure S1**). No 16S rRNA genes were amplified in the sterile controls; indicating that these remained sterile throughout the entire incubation even though lids were ajar to allow air exchange.

### Microbial Community Analysis

**FIGURE 2:**
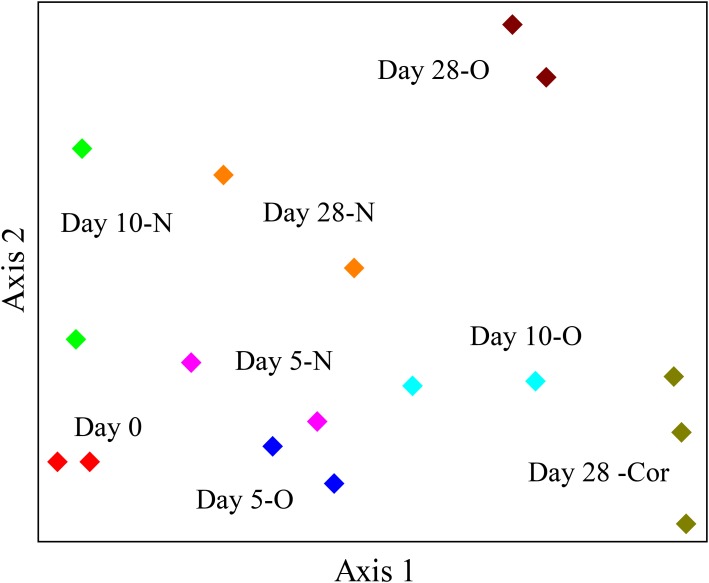
NMS ordination of bacterial sequences in the offshore experiment. Bottles contained seawater (800 mL), and either no amendment (N; biotic control), oil (O; 15 mg/L), or Corexit 9500 (Cor; 15 mg/L). Bottles were incubated at 2°C for 0 days (red), 5 days (N = pink; O = blue), 10 days (N = green; O = turquoise), and 28 days (N = orange; O = brown; Cor = dark green).

The response of indigenous microbial communities to ANS crude oil and Corexit 9500 was monitored over the course of the incubations in order to identify potential oil- and Corexit-degrading bacteria in Arctic seawater. The 16S rRNA gene amplicon sequencing results of all samples combined produced an average of 23,836 different OTUs. In the offshore incubation, the dendrogram and the NMS ordination both illustrated a strong separation in bacterial community structure between oil and Corexit incubations at day 28 (**Figure 2** and **Supplementary Figure S2**). In the offshore incubation, only two replicate mesocosms were incubated for the majority of treatments analyzed for microbial analyses due to limited seawater availability; however, these duplicates showed consistent grouping in our dendrogram and our NMS ordination (**Figure 2** and **Supplementary Figure S2**). Consistent grouping was also observed in the ordination with near-shore seawater, where bacterial sequences from replicate incubations grouped together and control incubations (seawater in the absence of Corexit) grouped separately from Corexit treatments (**Supplementary Figure S3**).

Multi-response permutation procedure was conducted to determine if microbial community shifts over the course of Corexit incubations were statistically significant. At each time point, the microbial communities were significantly different and they were significantly altered by the presence of Corexit (**Supplementary Figure S3**; MRPP, *p*-value < 0.05). Even though the structures of the microbial communities were drastically different (**Figure 1**; MRPP, *p*-value < 0.05), the biotic control at day 10 contained a similar abundance of prokaryotes as the Corexit treatment at day 10 in the near-shore seawater.

### Taxa That Responded to Oil or Corexit

**FIGURE 3:**
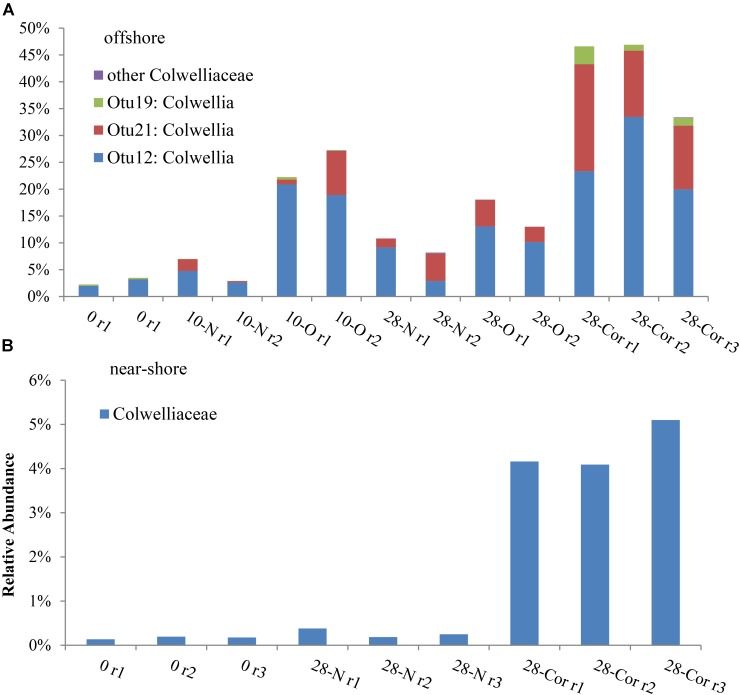
Relative abundance of bacterial sequences classified in the Colwelliaceae family in the **(A)** offshore and **(B)** near-shore experiment (2°C). Bottles contained seawater (800 mL), and either no amendment (N), oil (O; 15 mg/L), or Corexit 9500 (Cor; 15 mg/L). Individual OTUs (i.e., bacterial sequences) are identified in the offshore experiment to provide a specific comparison between oil and Corexit treatments.

In the offshore incubation, *Colwellia* (**Figure 2** and **Supplementary Figure S4**), *Oleispira* (**Supplementary Figures S4, S5a**), *Lutibacter* (**Supplementary Figure S6a**), and an unclassified member of Flavobacteriaceae (OTU83; **Supplementary Figure S6a**) increased in relative abundance in response to both oil and Corexit. One *Oleispira* OTU (OTU8) increased in relative abundance in response to oil at day 5 by 18% and day 10 by 14% (based on only 1 replicate in the latter case), and in response to Corexit at day 28 compared to the biotic controls (*n* = 2; **Supplementary Figure S5a**). In addition, one *Colwellia* OTU (OTU12) increased in response to oil at day 10 (by 16%), oil at day 28 (by 8%), and Corexit at day 28 (by 22%) compared to the biotic control (**Figure 3A**). Overall, the relative abundance of *Colwellia* increased in response to oil (within 10 days) and increased in response to Corexit (between 10 and 28 days) in the offshore incubation; however, more *Colwellia* grew in response to Corexit than oil (relative abundance, **Figure 3A**).

All OTUs identified as indicator species using ISA were also found to increase in relative abundance compared to the biotic control. In the offshore incubation, three *Colwellia* OTUs (OTUs 12, 19, and 21) and an individual unclassified Flavobacteriaceae (OTU83) were identified as indicator species for the presence of Corexit at day 28, with indicator values and *p*-values of IV = 88, *p* = 0.013; IV = 67, *p* = 0.012; IV = 56, *p* = 0.013; and IV = 88; *p* = 0.013, respectively. In response to oil-only, *Sulfitobacter* (OTU72), an unclassified Flavobacteriaceae (OTU50), and an unclassified Rhodobacteraceae (OTU4) increased in relative abundance compared to the biotic controls within 28 days in offshore incubations (**Supplementary Figure S7**). ISA also identified an individual *Sulfitobacter* (OTU72) as an indicator species for the presence of oil at day 28 (IV = 88; *p* = 0.013). All indicator species identified in incubations at day 0 and day 28 are shown in **Supplementary Table S2**.

In the near-shore experiment, members of Oceanospirillaceae, Flavobacteriaceae, and *Colwellia* also increased in response to Corexit compared to the biotic controls. *Polaribacter*, a member of Flavobacteriaceae, increased in relative abundance in response to Corexit at day 10 (by 34%; **Figure 1** and **Supplementary Figure S6b**), while Oceanospirillaceae (**Supplementary Figure S5b**) and Colwelliaceae (**Figure 3B**) increased in response to Corexit at day 28.

### Functional Gene Analysis

**FIGURE 4:**
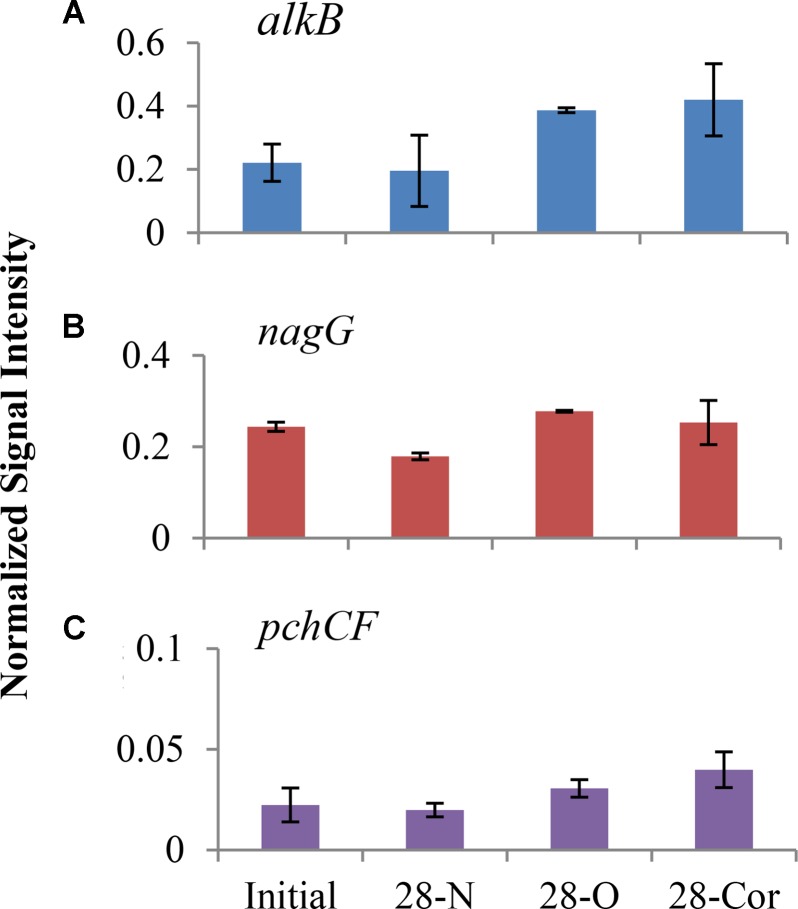
Normalized signal intensity of *alkB* **(A)**, *nagG* **(B)**, and *pchCF* **(C)** genes in offshore experiment at day 0 and 28 (2°C; n = 3). Bottles contained seawater (800 mL), and either no amendment (N), oil (O; 15 mg/L), or Corexit 9500 (Cor; 15 mg/L). Error bars are standard deviations.

We used GeoChip analyses to detect and compare abundances of genes that encode microbial enzymes used in biodegradation to identify metabolic processes potentially utilized by oil- and Corexit-degrading microorganisms in offshore surface seawater. At 28 days, the normalized intensities of total petroleum degradation genes (*alkB*, *apc*, *bbs*, *catB*, *ebdA*, *edbABC*, *hbh*, *pchCF*, *tomoABE*, *tamA*, *tutFDG*, *xylM*, *catA*, multi-ring-1,2-dixoygenase, one-ring-1,2-dioxygenase, *nagG*, one-ring-2,3-dioxygenase) in the biotic control (day 28N) grouped separately from treatments amended with oil or Corexit in the NMS ordination, and a slight separation was observed between oil and Corexit incubations (**Supplementary Figure S8**). The signal of several individual genes did shift over the course of incubation with oil or Corexit (**Figure 4**). At day 28, *alkB* (alkane monooxygenase), *nagG* (salicylate 5-hydroxylase), and *pchCF* (p-hydroxybenzaldehyde dehydrogenase) genes showed the greatest differences in signal intensity in both treatments compared to the biotic control (**Figure 4**). The relative abundance of *catA* (catechol dioxygenase), *catB* (muconate cycloisomerase), and to a smaller extent *one-ring-2,3-diox* (aromatic-ring-2,3-dioxygenase) increased in response to oil, but did not increase in response to Corexit. In the biotic control (seawater without oil or Corexit), the richness of *alkB* decreased from 67 different genes to 32 within the first 28 days. In the presence of oil or dispersant, the richness of *alkB* increased from 67 different genes to 119 in the oil treatment and to 179 in the Corexit treatment.

## Discussion

We report here that crude oil and surfactant components of Corexit 9500 can undergo significant biodegradation in Arctic surface seawater at 2°C. In near-shore and offshore seawater, respectively, the indigenous microbial community biodegraded 36% and 41% of oil, and 33% and 77% of the ionic surfactant DOSS within 28 days (**Tables 1**, 2). With respect to the non-ionic surfactants, the communities substantially biodegraded Span 80, while the majority of Tweens were lost due to abiotic processes. Microbial community shifts in response to oil or Corexit implicated several taxa in their biodegradation; with *Colwellia* and *Oleispira* having the strongest response to oil, and *Colwellia* and *Polaribacter* responding strongly to Corexit. Overall, Corexit appeared to enrich a different microbial community than crude oil in offshore surface seawater at 28 days (**Figure 2** and **Supplementary Figure S2**); however, several taxa (*Oleispira*, *Colwellia*, *Lutibacter*, and an unclassified Flavobacteriaceae spp. OTU83; **Figures 1, 3** and **Supplementary Figures S4, S5a**) and functional genes associated with oil biodegradation (*alkB*, *nagG*, and *pchCF*; **Figure 4**) increased in response to both oil and Corexit. These results suggest that some oil-degrading bacteria may also have the potential to biodegrade components in Corexit.

Whole crude oil biodegraded at a rate of 0.010 day^-1^ and 0.014 day^-1^ in offshore Arctic seawater collected in September and October, respectively. While there is a lack of literature reporting whole oil biodegradation rates in Arctic marine environments (National Research Council [NRC], 2014), these rate constants are aligned with recent reports of physically and chemically dispersed oil at temperatures ranging from -1°C to 13°C in the absence of nutrients. In this experiment, we did not directly compare offshore oil degradation to that occurring near shore, but in a previous mesocosm experiment run under similar conditions with near-shore Arctic seawater, ANS crude oil (2.5 mg/L), and no added nutrients, McFarlin et al. (2014) measured the loss of total detectable hydrocarbons and reported a half-life of 40 days (Prince et al., 2017) at -1°C, which equates to a first-order rate constant (*k*) of 0.017. In addition, Ribicic et al. (in press) also measured the loss of total detectable hydrocarbons in incubations containing chemically dispersed oil (25 μm droplets; 2–3 mg/L) and no added nutrients. Within 64 days, Grane oil biodegraded at a rate of 0.0095 day^-1^ (at 5°C) and at a rate of 0.0113 (at 13°C).

The influence of temperature on oil biodegradation rates has been extensively studied (Atlas and Bartha, 1972; Atlas, 1981; Brakstad et al., 2008) and reduced losses at low temperatures are often reported. Recent research suggests that physico-chemical properties of oil at low temperature are more likely to limit oil biodegradation than reduced metabolic rates (Bagi et al., 2013). While it is interesting to note that biodegradation rates may be surprisingly similar across temperatures and regions, the combination of biodegradation and abiotic losses are the most relevant to predicting the fate of oil in the environment. Such comparative studies show lower extents of oil loss in the Arctic (McFarlin et al., 2014) than in temperate regions (Prince et al., 2013) within the same time period (**Table 1**). Abiotic factors, such as evaporation and diffusion, increase with increasing temperature (Honrath and Mihelcic, 1999). Therefore, more total oil is lost in temperate (Prince et al., 2013) vs. Arctic environments (McFarlin et al., 2014) when abiotic losses are combined with biodegradation.

Significant losses (abiotic + biotic) of the surfactant constituents of Corexit occurred (35–98% loss of DOSS, >97% of Span 80, >99% of Tweens; *p* > 0.05) within 28 days in both offshore and near-shore incubations (**Table 2**). Abiotic loss of the surfactant components of Corexit varied considerably, with Tweens experiencing the greatest abiotic loss. Complete loss (to below LOD) of Tweens was observed within 10 days (**Table 2**), which was also observed by Kleindienst et al. (2015) in a laboratory microcosm study using Corexit 9500 added (alone) to Gulf of Mexico deep-seawater at 8°C. Degradation rates of DOSS have yet to be reported in other cold environments; however, the biodegradation of DOSS (33–77% over 28 days) observed in our study (2°C) is in contrast to Kleindienst et al. (2015) who reported an 8% loss of DOSS over 28 days in their Corexit-only incubation at 8°C. When Corexit was incubated together with oil, in the form of chemically enhanced water accommodated fractions (CE-WAFs), Kleindienst et al. (2015) reported that 30% of DOSS biodegraded within 28 days. The differences in the extents of DOSS biodegradation observed in our studies compared to those of Kleindienst et al. (2015) may be a function of different microbial communities between the Gulf of Mexico and the Arctic and/or differing methodologies; e.g., direct addition of Corexit/oil vs. water accommodated fractions and our use of aerated mesocosms (stirred with lids ajar) containing freshly collected seawater compared to their use of sealed bottles on roller tables using seawater stored for over 1 month.

Other than our study, Campo et al. (2013) includes the only other published DOSS biodegradation rate constant in the absence of oil. In microcosms (100 mL) containing artificial seawater and cultured microorganisms from the Gulf of Mexico incubated at 25°C, Campo et al. (2013) reported that DOSS biodegraded to extents >99% after 8 days with a first-order rate constant of 0.30 day^-1^. Here, we report a substantially lower rate of DOSS biodegradation in Arctic seawater at 2°C, with a first-order rate constant of 0.015 day^-1^. Since only three time-points (day 0, 10, 28) were used in our experiments to calculate the rate of DOSS biodegradation, future experiments should be conducted with additional time points. Techtmann et al. (2017) used the same culture as Campo et al. (2013) and also reported a rapid degradation of DOSS at 25°C, but no degradation at 5°C. Our experimental methods were substantially different from those used by Campo et al. (2013) and Techtmann et al. (2017), which reduces the comparability of our results. They used cultures rather than intact seawater communities and their cultures were enriched with oil as the only carbon source; and thus conducted Corexit-only biodegradation experiments with cultures selected for oil degradation. In the presence of oil, Techtmann et al. (2017) reported that DOSS was completely degraded; suggesting that oil enhances the biodegradation of DOSS.

We observed differences in the extent of DOSS biodegradation between our near-shore and offshore seawater incubations (33 ± 7% loss vs. 77 ± 0.5% loss, respectively; **Table 2**), which were both conducted at the same temperature (2°C), suggesting that variables like microbial community structure may affect DOSS biodegradation. The offshore location is characterized by Bering Sea water flowing north from the Bering Strait, and the near-shore location is characterized by coastal water that flows northward via the Alaskan Coastal Current (Day et al., 2013). Greater DOSS biodegradation occurred in offshore (77 ± 0.5%) than near-shore (33 ± 7%) incubations in 28 days (**Table 2**), which was also correlated with a greater response of *Colwellia* (42% of the community in offshore seawater at day 28 vs. 4% of the community in near-shore; **Figure 1**). At day 0, *Colwellia* was also present at a greater relative abundance in the offshore incubation (3% of the total community) than the near-shore incubation (0.2%). While *Colwellia* was the genus that increased in abundance the most in the offshore incubations; in the near-shore incubations, *Polaribacter* showed the greatest change when it increased by 30% within the first 10 days (**Figure 1**). Within our dataset, psychrotrophs *Colwellia* and *Polaribacter* (Deming and Junge, 2005; Moyer and Morita, 2007) are likely the most influential in the biodegradation of Corexit in Arctic seawater. These findings also indicate that the extent of Corexit biodegradation in Arctic seawater is determined by microbial properties that may vary with season and geographic location.

*Colwellia* may have a greater role in degrading Corexit than crude oil in Arctic marine environments. In offshore seawater at day 28, *Colwellia* spp. (OTUs 12, 19, and 21) were indicators of the presence of Corexit (ISA), and incubations containing Corexit had a higher relative abundance of *Colwellia* (**Figure 3A**) and total prokaryotes (**Figure 1** and **Supplementary Figure S1**) than incubations containing oil. The genus *Colwellia* is known to include psychrophilic species isolated from deep sea and polar marine ice (Deming and Junge, 2005), and have been associated with the biodegradation of oil in Antarctic seawater cultures (Yakimov et al., 2003), Arctic marine ice (Brakstad et al., 2008), and sub-Arctic seawater (Brakstad and Bonaunet, 2006). *Colwellia* spp. were identified as dominant members in a deep-water dispersed plume during the DWH oil spill and in enrichment incubations containing chemically dispersed oil in water from the Gulf of Mexico (Baelum et al., 2012; Chakraborty et al., 2012; Redmond and Valentine, 2012; Mason et al., 2014; Kleindienst et al., 2015). *Colwellia* spp. have also been shown to incorporate ^13^C from ethane, propane, and benzene at 6°C in stable isotope experiments (Redmond and Valentine, 2012) and grow on MC252 oil as the sole carbon source at 5°C (Dubinsky et al., 2013). Different *Colwellia* strains have the genetic potential to biodegrade a variety of hydrocarbons (gaseous, aromatics, n-alkanes, and cycloalkanes; Techtmann et al., 2016), which may be due to their acquisition of different degradative pathways through horizontal gene transfer (Collins and Deming, 2013). The increased relative abundance of *Colwellia* in our incubations with Corexit (**Figure 3**) together with the increase in total prokaryotic abundance (**Supplementary Figure S1**) supports prior reports of their rapid response to dispersed oil in temperate environments (Kleindienst et al., 2015) and labile carbon substrates in Arctic environments (Collins and Deming, 2013).

The abundance of *Polaribacter* coincided with the biodegradation of non-ionic surfactant components of Corexit at day 10 (**Figure 1** and **Table 2**) and may indicate growth on these components. *Polaribacter* spp. have also been found to increase in abundance in response to oil in sub-Antarctic seawater cultures (Prabagaran et al., 2007) and mesocosms consisting of Arctic sea ice (Garneau et al., 2016). *Polaribacter* spp. were also suggested to play a role in the degradation of complex organic matter in the deep-sea decaying microbial bloom in the aftermath of the DWH oil spill (Dubinsky et al., 2013).

Some microorganisms are known to use dispersants as growth substrates (Chakraborty et al., 2012). In our incubations, Corexit enriched a higher abundance of microorganisms than oil (**Figure 1** and **Supplementary Figure S1**), which was also observed by Lindstrom and Braddock (2002) and Kleindienst et al. (2015). When oil is chemically dispersed, laboratory studies have shown that oil-degrading microorganisms rapidly colonize dispersed oil droplets (Macnaughton et al., 2003), and may preferentially degrade some dispersant compounds over oil compounds (Foght and Westlake, 1982; Bunch et al., 1983; Foght et al., 1983). We previously reported (McFarlin et al., 2014) that indigenous Arctic marine microorganisms mineralized more Corexit than crude oil (20% weathered) continuously throughout a 60-day respirometer experiment at subzero temperatures (-1°C). Combined with the results of the present study, this suggests that microorganisms more readily utilize Corexit as a growth substrate than crude oil.

This study revealed that several taxa known to include oil degraders may also have the ability to biodegrade components in Corexit. We also observed that known oil degradation genes, most notably *alkB* and *nagG*, increased in intensity (based on the GeoChip) in both oil and Corexit incubations (**Figure 4**). The *nagG* gene encodes salicylate-5-hydroxylase, an enzyme that converts salicylic acid to gentisic acid, which is ultimately degraded to pyruvic and fumaric acid (Fuenmayor et al., 1998). Alkane monooxygenases (*alkB*) hydroxylates alkanes to alcohols (Rojo, 2009), and are the most common alkane hydroxylating enzymes found in bacteria (Smits et al., 1999, 2002). The increased abundance of *alkB* in Corexit incubations may be coincidental, but it is conceivable that these genes may assist in the biodegradation of alkanes in the petroleum distillate fraction of Corexit or the hydrocarbon side chains of the surfactants.

Together, our results indicate that surfactant components of Corexit 9500 undergo substantial biodegradation in Arctic seawater and that *Colwellia, Polaribacter, Oleispira, and other taxa* are implicated in this process. We also determined that both *Colwellia* and *Oleispira* are likely to play a significant role in oil biodegradation in Arctic seawater and report oil biodegradation rates of 0.010 day^-1^ and 0.014 day^-1^ in offshore seawater collected in September and October, respectively. The specific taxa that responded most to Corexit varied depending on the location and timing of seawater sampling, suggesting that differences in community structure in different water masses may lead to different responses. Taxa that responded to oil or Corexit in our Arctic seawater incubations also increased in abundance in response to the cold deep-water plume during the DWH oil spill (i.e., *Colwellia* and *Oleispira*; Redmond and Valentine, 2012; Dubinsky et al., 2013), supporting the ubiquitous nature of these oil-degrading microorganisms. Interestingly, some organisms and oil biodegradation genes (e.g., *alkB*) increased in relative abundance in response to both oil and Corexit, suggesting that some organisms may be capable of biodegrading components of both oil and Corexit. While this study provides a glimpse at Corexit 9500 biodegradation when present alone in Arctic seawater, such as in the case of off-target dispersant application, future studies should investigate the microbial response oil and Corexit when present together in Arctic seawater to better understand their fate and interactions in the context of a dispersed oil plume.

## Notes

The work was conducted at the University of Alaska Fairbanks and Oregon State University.

## Data Availability

Data are publicly available through the Gulf of Mexico Research Initiative Information and Data Cooperative (GRIIDC) at https://data.gulfresearchinitiative.org (doi: 10.7266/N7XW4GWQ). This is ECOGIG contribution number 479.

## Author Contributions

KM conducted the experimental design, the experimental set up and sampling, all DNA extractions, Corexit extraction, analyzed the 16S rRNA data set, analyzed the GeoChip data set, analyzed the GC/MS data, and wrote the paper. MP conducted the Corexit analysis, including the design of the extraction method, running the samples on the LC/MS/MS, and analyzing the data. MP also assisted in editing the entire manuscript and writing the Corexit analysis methods. JF provided mentorship regarding the Corexit analysis and contributed to its corresponding experimental design, data analysis, reporting, and discussion, also assisted in editing the entire manuscript. ML served as the principle investigator of the project, mentored KM, and was highly involved in the writing.

## Conflict of Interest Statement

The authors declare that the research was conducted in the absence of any commercial or financial relationships that could be construed as a potential conflict of interest.

## Footnotes

1 http://www.ou.edu/ieg/tools/data-analysis-pipeline
