# Supplementary material for: Biodegradation of Crude Oil and Corexit 9500 in Arctic Seawater
Source: Front Microbiol. 2018 Aug 6;9:1788. doi: 10.3389/fmicb.2018.01788 (PMC6096335; doi:10.3389/fmicb.2018.01788)
Supplement: Supplementary file 1 [file Data_Sheet_1.docx]

## Supporting Information

**Tables**

Table S1. Experimental design of mesocosm incubations containing 800 mL of seawater in 1 L flasks. Experiments are identified by the month and location of seawater collection and identify the carbon substrate tested (oil or Corexit) in parentheses. Time points are included under each analysis. All chemical analyses were conducted with abiotic controls and abiotic losses were subtracted. All microbial analyses were conducted with seawater-only controls to monitor the microbial community and gene presence in the absence of oil or Corexit.

| **Experiment** | **Oil analysis**  **Time points** | **Corexit analysis**  **Time points** | **Microbial analysis**  **Time points** |
| --- | --- | --- | --- |
| September offshore  (oil-alone) | day: 0,5,10,28 | no Corexit added | no microbial analyses |
| October offshore  (oil- and Corexit-alone) | day: 0,5,10,28 | day: 0 & 28 | day: 0, 5-oil*,10-oil*,28-oil*, 28-Corexit |
| August near-shore  (Corexit-alone) | no oil added | day: 0,10,28,60 | day: 0,10,28,60 |

* only two replicates were included for the microbial analyses on day 5, day 10, and day 28 with oil (-oil) for the October offshore experiment.

**Table S2.** Indicator species analysis (ISA) results for the offshore incubation. Indicator values (IV) represent the relative abundance and constancy of each OTU and range from 0 (no indication) to 100 (perfect indication). The statistical significance of each IV is indicated by a *p*-value and only significant (*p* < 0.05) IVs are present. Day 0 represents the initial seawater.

| **Day 0** |  |  |  |  |  |  |  |
| --- | --- | --- | --- | --- | --- | --- | --- |
| OUT | IV | *p*-value | Phylum | Class | Order | Family | genus |
| OTU9 | 97 | 0.014 | Proteobacteria | Gammaproteobacteria | unclassified | unclassified | unclassified |
| OTU588 | 87 | 0.037 | unclassified | unclassified | unclassified | unclassified | unclassified |
| OTU729 | 79 | 0.048 | Proteobacteria | Alphaproteobacteria | Rhizobiales | Brucellaceae | *Ochrobactrum* |
| OTU14 | 72 | 0.023 | Proteobacteria | Gammaproteobacteria | Alteromonadales | Pseudoalteromonadaceae | *Pseudoalteromonas* |
| OTU114 | 67 | 0.029 | Proteobacteria | Gammaproteobacteria | Pseudomonadales | Pseudomonadaceae | *Pseudomonas* |
| **Day 28-No addition** | |  |  |  |  |  |  |
| OTU266 | 98 | 0.013 | Proteobacteria | Gammaproteobacteria | unclassified | unclassified | unclassified |
| OTU45 | 70 | 0.023 | Proteobacteria | Gammaproteobacteria | unclassified | unclassified | unclassified |
| OTU16 | 57 | 0.032 | Proteobacteria | Gammaproteobacteria | Thiotrichales | Piscirickettsiaceae | *Methylophaga* |
| **Day 28-Oil** | |  |  |  |  |  |  |
| OTU1033 | 91 | 0.010 | Bacteroidetes | Flavobacteriia | Flavobacteriales | unclassified | unclassified |
| OTU72 | 88 | 0.013 | Proteobacteria | Alphaproteobacteria | Rhodobacterales | Rhodobacteraceae | *Sulfitobacter* |
| OTU117 | 84 | 0.026 | Proteobacteria | Gammaproteobacteria | Gammaproteobacteria | Arenicella | unclassified |
| OTU1165 | 77 | 0.039 | Bacteroidetes | unclassified | unclassified | unclassified | unclassified |
| OTU201 | 77 | 0.035 | Proteobacteria | Gammaproteobacteria | Alteromonadales | Alteromonadaceae | *Glaciecola* |
| OTU466 | 71 | 0.025 | unclassified | unclassified | unclassified | unclassified | unclassified |
| OTU49 | 65 | 0.026 | Bacteroidetes | unclassified | unclassified | unclassified | unclassified |
| OTU129 | 65 | 0.042 | Proteobacteria | Alphaproteobacteria | Sphingomonadales | Sphingomonadaceae | unclassified |
| **Day 28-Corexit** | |  |  |  |  |  |  |
| OTU156 | 100 | 0.013 | Proteobacteria | Alphaproteobacteria | Rhodobacterales | Rhodobacteraceae | unclassified |
| OTU246 | 97 | 0.013 | Proteobacteria | Gammaproteobacteria | Oceanospirillales | Oceanospirillaceae | unclassified |
| OTU236 | 93 | 0.013 | Proteobacteria | Gammaproteobacteria | unclassified | unclassified | unclassified |
| OTU19 | 88 | 0.013 | Proteobacteria | Gammaproteobacteria | Alteromonadales | Colwelliaceae | *Colwellia* |
| OTU75 | 83 | 0.041 | Bacteroidetes | Flavobacteriia | Flavobacteriales | Cryomorphaceae | unclassified |
| OTU83 | 74 | 0.013 | Bacteroidetes | Flavobacteriia | Flavobacteriales | Flavobacteriaceae | unclassified |
| OTU101 | 69 | 0.013 | Proteobacteria | Alphaproteobacteria | Rhodobacterales | Rhodobacteraceae | *Loktanella* |
| OTU21 | 67 | 0.012 | Proteobacteria | Gammaproteobacteria | Alteromonadales | Colwelliaceae | *Colwellia* |
| OTU12 | 56 | 0.013 | Proteobacteria | Gammaproteobacteria | Alteromonadales | Colwelliaceae | *Colwellia* |

**Figures**

a

c

b

d

**NM**

**NM**

b

b

a

**NM**

### Figure S1. Mean abundance of prokaryotes in offshore and near-shore experiments at 2°C. Bottles contained seawater (800 mL), nutrients (16 mg/L Bushnell Haas), and either no amendment (N; biotic control), oil (O; 15 mg/L), or Corexit 9500 (Cor; 15 mg/L). Different letters correspond to significant differences among treatments containing three replicates (MRPP, *p* < 0.05). NM: not measured.

### Figure S2. Dendrogram of bacterial sequences (16S rRNA genes) in offshore experiment. Bottles were incubated at 2°C for 28 days and contained offshore seawater (800 mL), nutrients (16 mg/L Bushnell Haas), and either no amendment (N; green), oil (15 mg/L; blue), or Corexit 9500 (C; 15 mg/L; orange). Day 0 is the initial seawater prior to any addition (red).

Day 10-N

Day 10-Cor

Day 0

Day 28-N

Day 28-Cor

### Figure S3. NMS ordination of bacterial sequences in the near-shore experiment (*n* = 3). Bottles contained seawater (800 mL), nutrients (16 mg/L Bushnell Haas), and either no amendment (N; biotic control), or Corexit 9500 (Cor; 15 mg/L), and were incubated for 0 (red), 10 (Cor= turquoise; N = green), and 28 days at 2°C (Cor = blue; N = pink).

### Figure S4. Relative abundance of bacterial genera in the offshore experiment. Bottles contained seawater (800 mL), nutrients (16 mg/L Bushnell Haas), and either no amendment (N; biotic control), oil (O; 15 mg/L), or Corexit 9500 (Cor; 15 mg/L), and were incubated for 0, 5, 10, and 28 days at 2°C. The Corexit treatment (28C, *n* = 3) was only incubated for 28 days in the offshore experiment.

(b) near-shore

(a) offshore

### Figure S5. Relative abundance of bacterial sequences classified in the Oceanospirillaceae family at day 0 and 28 in the (a) offshore experiment and (b) near-shore experiment. Bottles contained seawater (800 mL), nutrients (16 mg/L Bushnell Haas), and either no amendment (N), oil (O; 15 mg/L), or Corexit 9500 (Cor; 15 mg/L) and were incubated at 2°C. Individual OTUs (sequences) are identified in the October offshore experiment to provide a specific comparison between oil and Corexit treatments.

(b) near-shore

(a) offshore

Figure S6. Relative abundance of bacterial sequences classified in the Flavobacteriaceae family in the (a) offshore experiment and (b) near-shore experiment at day 0, 10, and 28. Bottles contained seawater (800 mL), nutrients (16 mg/L Bushnell Haas), and either no amendment (N; biotic control), oil (O; 15 mg/L), or Corexit 9500 (Cor; 15 mg/L) and were incubated at 2°C. Individual OTUs (sequences) are identified in the offshore experiment to provide a specific comparison between oil and Corexit treatments.

### Figure S7. Relative abundance of sequences classified in the Rhodobacteraceae family at day 0 and 28 in incubations containing offshore seawater (800 mL), nutrients (16 mg/L Bushnell Haas), and either no amendment (N), oil (O; 15 mg/L), or Corexit 9500 (Cor; 15 mg/L) at 2°C. Individual OTUs (sequences) are identified to provide a specific comparison between oil and Corexit treatments.

### Figure S8. NMS ordination of petroleum degradation genes in offshore experiment. Bottles were incubated at 2°C for 28 days and contained offshore seawater (800 mL), nutrients (16 mg/L Bushnell Haas), and either no amendment (N; pink), oil (15 mg/L; turquoise), or Corexit 9500 (C; 15 mg/L; green). Day 0 is the initial seawater prior to any addition (red).
